# Research training needs in Peruvian national TB/HIV programs

**DOI:** 10.1186/1472-6920-10-63

**Published:** 2010-09-28

**Authors:** Patricia J Garcia, Armando Cotrina, Eduardo Gotuzzo, Elsa Gonzalez, Anne L Buffardi

**Affiliations:** 1 Epidemiology, STI and HIV Unit, School of Public Health and Administration, Universidad Peruana Cayetano Heredia, Lima, Peru; 2 Instituto de Medicina Tropical Alexander von Humboldt, Universidad Peruana Cayetano Heredia, Lima, Peru; 3 University of Washington Center for AIDS & STD, Seattle, Washington, USA

## Abstract

**Background:**

There are few published reports of *research training *needs assessments and research training programs. In an effort to expand this nascent field of study and to bridge the gap between research and practice, we sought to systematically assess the research training needs of health care professionals working at Peruvian governmental institutions leading HIV and tuberculosis (TB) control and among senior stakeholders in the field.

**Methods:**

Six institutional workshops were conducted with the participation of 161 mid-level health professionals from agencies involved in national HIV and TB control. At each workshop informants completed a structured questionnaire and participated in small and large group discussions. Additional data and institutional commitment was obtained through in-depth interviews from 32 senior managers and researchers from the Ministry of Health, academia and NGOs.

**Results:**

Participants exhibited an overwhelming receptivity for additional research training, observing a gap between current levels of research training and their perceived importance. Specialized skills in obtaining funding, developing research protocols, particularly in operational, behavioral and prevention research were considered in greatest need. Beyond research training, participants identified broader social, economic and political factors as influential in infectious disease control.

**Conclusions:**

The needs assessment suggests that future training should focus on operational research techniques, rather than on clinical skill building or program implementation only. Strengthening health systems not only requires additional research training, but also adequate financial resources to implement research findings.

## Background

Within the last decade, there has been growing international recognition of the central importance of strong health systems in enhancing health and reducing poverty, coupled with increases in funding to achieve these goals. The Global Forum for Health Research has highlighted the crucial role of research in such efforts to strengthen health systems, both the application of existing research, as well as the need for new research better targeted towards preventable health problems that disproportionately affect billions of people living in low and middle income countries. This dual strategy aims to reduce what the WHO terms the 'know-do gap' so that research findings can be translated into health policy and practice. It also requires health research capacity building and training across disciplines, particularly within countries that experience the highest burdens of disease [1,2].

Although there is an extensive literature on training health care providers to respond to infectious diseases epidemics, there are comparably fewer published reports of *research training *needs assessments and research training programs. In his recent review of the literature on medical research training programs, Barton critiques previous accounts for their lack of rigor and infrequent use of mixed methods that are necessary to provide sufficient depth of information to systematically evaluate such programs [3]. Existing research training programs have identified the need for and have employed a range of training modalities from a several hours seminar, to multi-year advanced degree training [3-5]. Nearly all have emphasized the importance of maintaining practitioners' base in the field and in their home country, while simultaneously providing protected work time during which to implement research under supervised mentorship [3,4,6,7]. Goding, Chew and Whitworth identify broader challenges specific to resource-constrained settings, including inadequate funding for health research, poor institutional infrastructure, and lack of appropriate research training, career development pathways and role models opportunities for research scientists [8].

In an effort to expand the nascent field of study of research training and to bridge the gap between research and practice, we sought to systematically assess the research training needs of Peruvian health care professionals employed predominantly as mid-level managers at public institutions leading HIV and TB diagnosis, prevention, treatment and care in Peru. As background context, Peru is currently experiencing rising incidence of multi-drug resistant tuberculosis (MDR TB), with 6% of the 1,989 confirmed MDR-TB cases reported from 1997 to 2007 classified as extensive drug-resistant tuberculosis (XDR-TB) [9]. Peru's HIV epidemic is concentrated in men who have sex with men, for whom prevalences range from 5.7% in a jungle city to 22.3% in Lima, the country's capital [10-12]. This research training needs assessment coincided with an influx of external resources to address infectious disease epidemics, in particular the Global Fund to Fight AIDS, Tuberculosis and Malaria. The expansion of health care services thus raises additional challenges regarding the scale-up of existing programs and the capacity of the current healthcare workforce to implement them.

With the ultimate aim of improving population-level health, the objectives of this training needs assessment were twofold: to identify the perceived needs by focal area, in relation to existing knowledge levels, and subsequently to prioritize these needs, recognizing the existence of finite resources with which to implement training programs [13]. The impetus for the needs assessment was to inform the development of a tailored training program, which has since been implemented.

## Methods

### Study population

From September 2003 to February 2004, we conducted several workshops where we administered structured questionnaires (Additional file 1) and facilitated group discussions to identify and prioritize research training needs among health care professionals working at health centers, hospitals or central offices of three governmental institutions: (1) the Peruvian Ministry of Health, including those whose responsibilities are related to the General Directorate of Epidemiology (DGE), the National STD/HIV/AIDS Control Program (ESNPETSS) and the National TB Control Program (ESNTB); (2) the Peruvian Social Security Health System (ESSALUD), including those from with responsibilities related to the Committee of Tuberculosis Control (TB ESSALUD) and the Committee of HIV/AIDS Control (HIV ESSALUD); (3) the Peruvian Armed Forces Health system, including those related to the HIV/AIDS Control and Prevention Program (COPRECOS) and those related to TB control (TB-FFAA). Together, these institutions represent the major public entities responsible for developing, coordinating and implementing the Peruvian government's public health strategies for TB and HIV control, and for conducting ongoing surveillance of these diseases. All needs assessment activities were conducted in Lima.

Participants were purposively selected from among staff in managerial or coordination roles, who were currently working directly on TB or HIV and who had worked in infectious disease control for at least one year. While responsible for running or coordinating activities related to HIV or TB control, these mid-level managers also had clinical responsibilities and treated patients as part of their routine activities at individual health centers and hospitals.

### Key informant interviews

Prior to conducting these institutional workshops, we first conducted key informant interviews with 32 senior stakeholders who had been working in the fields of TB and HIV for ten years or more.

In-depth interviews lasted approximately thirty to forty-five minutes each. Interviewees responded to seven open-ended questions that explored the principle challenges facing Peru regarding TB and HIV, potential solutions, principles of research, priorities for training in research and program management, and appropriate training modalities. These informants provided advice on how best to structure and who to involve in the institutional workshops, specifically recommending that we target mid-level managers who could provide dual clinical and managerial perspectives. In addition to providing these suggestions, these in-depth interviews also helped to engage key actors, who later facilitated access to their staff and whose buy-in is critical to the institutionalization of ongoing training programs.

### Survey instrument

We designed structured questionnaires with both open- and closed-ended questions based on discussions with key informants and a related literature search. The survey covered demographic characteristics, perceptions of barriers and facilitating factors in receiving training, and asked respondents to rank from 0 to 5 a) current level of training in Peru and b) importance of 51 different training topics covering nine broad themes: fundamentals of research, topics in epidemiology and biostatistics, specific topics related to HIV and TB research, behavior change intervention research, ethics, financing, publication, leadership, and planning, program implementation, monitoring and evaluation. We validated the questionnaire with 18 of our key informants.

### Institutional workshops

We next conducted six one day workshops with participants from health centers, hospitals, institutes and central offices working for the DGE, ESNPETSS, ESNTB, HIV ESSALUD, TB ESSALUD, COPRECOS and TB-FFAA. Participants first completed the structured questionnaire, after which the study team provided an overview of the needs assessment process. An official from the institution gave a formal presentation of the institution's current work in TB and/or HIV. In the afternoon, participants were divided into two groups HIV-related and TB-related, and then into small working groups, each tasked with responding to the open-ended questions, identical to those asked in the key informant interviews. The day concluded with a plenary session in which each small group shared their responses and the larger group was able to engage in discussions.

### Data analysis

Demographic data and rankings from the structured questionnaire were analyzed using SPSS 11.0 (Chicago, IL). We conducted content analysis of responses to the open-ended questions to identify prominent topical themes.

### Human subjects considerations

The needs assessment proposal was granted a certificate of exemption from the Institutional Review Board at Universidad Peruana Cayetano Heredia.

## Results

Results presented here represent responses to close and open-ended questions in the questionnaire, using qualitative responses from small and large group discussions and from key informant interviews to help clarify and contextualize the questionnaire responses.

### Participants characteristics

As presented in Table 1, 161 individuals participanted in the workshops. The group was gender balanced (49% female) and reflected the geographical headquarters of the institutions, with 76% of the sample based in Lima and the remaining 24% working in 18 smaller Peruvian provinces. Nearly two thirds of the respondents (60%) were trained in medicine and a quarter (25%) in nursing; the remaining participants represented dentistry, psychology, biology and social work. Few of the participants (26/161, 16%) reported having been involved in research.

**Table 1:** Characteristics of workshops' participants (N = 161)

Characteristics	N	%
**Gender**		
Male	89	51
Female	71	49
		
**Age**		
20-30 years	13	8
31-45 years	98	61
> 45 years	50	31
		
**Region**		
Lima	123	76
Other provinces	38	24
		
**Educational background**		
Medical doctor	96	60
Nursing	40	25
Midwives	6	4
Social Worker	5	3
Other (psychologist, dentist, biologist, other)	14	8
		
**Institutional affiliation**		
ESNETSS	33	20
ESNTB	32	20
HIV-DGE	15	9
TB-DGE	12	7
TB ESSALUD	29	18
HIV ESSALUD	20	12
COPRECOS	9	6
TB-FFAA	11	7

ESNPETSS = National STD/HIV/AIDS Control Program; ESNTB = National TB Control Program; HIV-DGE = General Directorate of Epidemiology, area of HIV, TB-DGE = General Directorate of Epidemiology, area of TB; TB ESSALUD = Peruvian Social Security Health System, Committee of Tuberculosis Control; HIV ESSALUD Peruvian Social Security Health System, Committee of HIV/AIDS Control; COPRECOS = Peruvian Armed Forces Health system, HIV/AIDS Control and Prevention Program; TB-FFAA Peruvian Armed Forces Health system TB control committee.

Key informants included the directors of DGE and ESNTB, chiefs of infectious diseases at several large hospitals, professors of medicine and epidemiology at Universidad Nacional Mayor de San Marcos and Universidad Peruana Cayetano Heredia, as well as directors of three non-governmental organizations (NGOs) involved in TB and HIV research and care. Forty one percent (13/32) reported previous involvement in research, less than half (6/13) of whom had authored at least one published research article.

### Receptivity to research training

Overall, there was wide acceptance of the importance of institutional capacity building and eagerness to take part in research training, with only 2 of 193 (1%) respondents indicating they would not be willing to take part in future training activities. Participants were in favor of a range of training options, demonstrating preferences for short (workshops or seminars) and medium (semester-long) training options compared to long-term (formal degree) training.

### Training needs

These health care professionals observed a gap between current levels of training, which ranged from medium to little knowledge about specific training topics, and the importance of these areas. Notably, all of the 51 training topics listed in the structured questionnaire were rated as important or very important, underscoring the enthusiasm for research training observed above. There was remarkable consistency in rankings between health care professionals working in TB and those in HIV, and between key informants and institutional participants, indicating that perceptions of training needs were neither disease-specific, nor dependent on the participants' status within the organization.

As illustrated in Table 2, respondents felt that the current healthcare workforce in Peru had the highest training levels in clinical topics and placed lesser importance on the need for additional training in immunology, viral and bacterial pathogenesis. Participants felt well trained in principles of biosafety and considered biosafety training to be highly important; conversely, they observed a lack of training in mathematical modeling, but perceived this topic to be less of a priority. Among research training areas considered of highest importance were prevention and behavioral research, operations research and program evaluation, strategies to identify and apply for research funding, protocol development.

**Table 2:** Perceived training needs among Peruvian TB & HIV health care professionals

Current training level		Importance of training on		
Highest rating				
1	Principles of infectious disease	1	Principles of biosafety	
2	Perinatal transmission of HIV	2	Prevention research, behavioral research	
3	Principles of biosafety	3	Program evaluation/operations research	
4	Global epidemiology of HIV/TB	4	Strategies to identify and apply for research funding, grant writing	
5	Principles of neumology	5	Protocol development, epidemiological design	
				
Lowest rating				
1	Mathematical modeling	1	Mathematical modeling	
2	Effective communication among investigators and national policymakers	2	Principles of immunology	
3	Prevention research/behavioral theories	3	Principles of bacterial pathogenesis	
4	Strategies to identify and apply for research funding	4	Principles of viral pathogenesis	
5	Principles of health economics	5	Immunology of HIV	

In addition to the list of 51 training topics presented in the questionnaire, respondents identified 175 unique research topics they considered important for future training, including multidrug resistant TB and anti-retroviral medications (regarding new drugs, costs, compliance, implementation of guidelines), and TB/HIV interactions. Analysis of responses to open-ended questions yielded similar results to the close-ended responses, with how to get funding and study design ranking among the highest importance.

### Barriers to engagement in research

In small and large group discussions, participants remarked on the paucity of research data on the national level that could be used to develop effective disease control program priorities. There was broad consensus that there exist few opportunities for research training for government health personnel within the current institutional structure, particularly in operational and health services research. These health care professionals cited time and money as primary barriers to their participation in research training activities, noting that their involvement was dependent on personal initiative and self-financing. They further commented on a lack of experience in implementing policies based on research findings, lack of research mentoring, and lack of opportunities for multidisciplinary research collaboration.

### Strengthening health systems and operational research

Open-ended responses elicited through the small group discussion indicated a strong interest in operational and health systems research, aimed at improving the quality of health care services provided in Peru. The specific terms "operational research" and "health systems research" were presented in the questionnaire. Some participants were already familiar with these terms through their interactions with international donors and NGOs engaged in operational research, while others described research questions that could be classified as operational and health systems research. As illustrated in Table 3, participants discussed the need to evaluate the impact of their programs, identify ways in which they could improve adherence with case management and treatment protocols, and examine how programs could best be managed to improve the quality of attention patients receive and to optimize resources. They indicated strong interest in program evaluation and the assessment of implementation efforts. Several expressed a desire for training in benefit-cost analyses.

**Table 3:** Qualitative responses from mid-level managers regarding research priorities in Peru

On the importance of research
"We need to convince high level authorities about the need to do research, good practical research to improve our services."

"We have to be trained to be able to talk to and convince our authorities [to undertake research], or we have to train our authorities to change."

"We need to do more evidenced-based public health activities. Research can provide us this evidence."
**On the need for operations research**
"I think that at this point what our country needs is - rather than general research or clinical research on individuals - research on how our health system works; that is, how it operates... operations research and how to help improve it..."

"We need more operations research, something that has been done by NGOs and international donor agencies, but it has been restricted to a few people who know how to do it and they don't even share the results. All health workers should know how to do it and should use operations research as a tool to improve their services."

"We have to learn how to develop research in teams, as part of our day-to-day work. This could become a permanent system to improve."

"I think that a priority is to learn basic epidemiology, but a much higher priority is to learn economics - cost effectiveness it is called, right? And how health systems work and can be improved."

"We should start teaching people about research methods and operations research, health services research, something that will be practical and useful."

### Broader socio-political and economical context affecting program results and research

In addition to responses to specific research topics, the key informant interviews and small group discussions highlighted broader socio-economic factors that affect TB and HIV diagnosis: poverty and a culture of silence surrounding HIV and TB and human rights concerns, particularly among vulnerable populations. Participants also discussed political and management issues, such as the weakening of the national TB and HIV programs both financially and politically in recent years, inconsistent political support due to frequent turnover of senior leadership, a lack of coordinated policies among entities engaged in infectious disease control, including between the public and private sectors, and weak ties with the broader community.

## Discussion & Conclusions

This research training needs assessment among mid-level health care professionals with dual managerial and clinical roles, and key informant interviews with senior leaders in the field demonstrates that there is a shared perception of the need for training in TB and HIV research and an overwhelming receptivity to take part in future training activities. The results suggest that training topics should focus less on clinical knowledge and more on specific research skills, such as protocol development and operational research design aimed at prevention and treatment. These findings support Dayrit et al's call for more operational and context-specific research conducted at a local country level under the umbrella of a national strategy of health research [14]. Barriers to research training identified by our respondents were consistent with other reports [3,6,7], highlighting the need for both supplemental sources of funding from external entities, as well as internal program changes to enable protected work time on research activities.

The satisfaction with clinical training expressed by our participants may in large part reflect the educational background of these health care professionals, who were disproportionately trained in medicine and nursing. This clinical training is focused on skill building and service delivery: diagnosing disease and treating patients. What appears to be lacking for mid-level managers are the *antecedents*, how to design research studies and public health interventions and obtain resources to fund them, and the *assessment *of current health care practices in the field. These findings are consistent with previous research training needs assessments that have reported gaps in experience at these two ends of the research spectrum [5,7]. Many of the participants working in the Ministry of Health National Strategy Programs for TB and HIV and our key informants could be considered policymakers, in that they are the ones developing national standards and regulations for TB and HIV prevention and treatment; however, in each of these groups, insufficient political support was cited as a barrier to effective infectious disease control.

Recognizing that the core function of these institutions is to provide health care services, resources may be best allocated toward providing background training in operational research methods and then creating links between these institutions and research scientists at universities and NGOs so these groups can jointly develop rigorous but feasible research studies, relevant to current issues in the field. Tailored research training would enable government providers to identify research questions and better understand how data can best be collected, but it may be outside the scope of their positions to undertake large research projects. For mid-level managers who are able to integrate research into their program portfolio, structured mentoring relationships with more experienced research scientists appears to be critical to sustained research engagement. In addition to the need for collaboration across institutions, research activities may be enhanced by creating more multidisciplinary teams that include biostatisticians, communications specialists, policy analysts and health economists to complement the predominance of medical expertise.

Although this needs assessment was centered on research training as one mechanism to improve infectious disease control in Peru, several of the themes raised in the open-ended responses suggest that the impact of expanded training opportunities may be context dependent. That is, health system improvements cannot rely on increased knowledge and technical skills alone; they also require ongoing political commitment, adequate financial resources, respect for human rights and poverty reduction efforts. In this sense, our needs assessment was both definitive and exploratory, in that it prioritized specific topics for future training, but also uncovered issues for which there is no easily implementable solution [13].

These findings largely reflect the perceptions of mid-level and senior managers working within the six national institutions that participated in the research training needs assessment. Since participants were nominated by the institutions themselves, this sample may be biased towards those with a greater affinity towards research or who may be more receptive to training. Participants from Lima were disproportionately represented, though the work of many of these health care professionals is national in scope, and those that work at health centers and hospitals in Lima serve one third of the Peruvian population that resides in the capitol city, where TB and HIV prevalences are much higher than national averages. These results do not reflect perceived needs of health care personnel engaged in private practice.

The way in which the needs assessment was conducted, through participatory workshops, served to foster deeper dialogue and reflection about the role of research within leading government institutions. However, engaging in group discussions may have tempered individual responses, which underscores the importance of triangulating data from self-administered questionnaires, individual interviews with key informants and small and large group discussions.

This research training needs assessment has already proved useful within Peru in guiding future training efforts and research activities for mid-level professionals. On a broader level, it offers insights that may be applicable to other national infectious disease control programs, in terms of the perceived gaps in research training, need for a more multidisciplinary approach, and consideration of contextual factors. These activities offered an initial opportunity to bridge the siloed spheres of academic research scientists and program manager-practitioners, for just as these two diseases gain strength through their interaction, so too must those engaged in their control.

## Competing interests

The authors declare that they have no competing interests.

## Authors' contributions

PJ conceived of and conducted the study, performed the analysis and helped to draft the manuscript. AC conducted the study and assisted in the analysis. EG conceived of and conducted the study. EG conducted the study. AB performed the analysis and drafted the manuscript. All authors read and approved the final manuscript.

## Pre-publication history

The pre-publication history for this paper can be accessed here:

http://www.biomedcentral.com/1472-6920/10/63/prepub

## Supplementary Material

Additional file 1 **Questionnaires**. Questionnaires used to collect information for the study (in Spanish). Click here for file
