# Supplementary material for: Research training needs in Peruvian national TB/HIV programs
Source: BMC Med Educ. 2010 Sep 28;10:63. doi: 10.1186/1472-6920-10-63 (PMC2955044; doi:10.1186/1472-6920-10-63)
Supplement: Additional file 1 — Questionnaires. Questionnaires used to collect information for the study (in Spanish). [file 1472-6920-10-63-S1.PDF]

Questionnaires used in the Needs Assessment ICORHTA PROGRAM for TB and HIV

1. Pilot instruments
2. Validated final instruments

ANEXOS

INSTRUMENTOS INICIALES (PILOTO)

ANEXOS: ENTREVISTA CLAVE

Estamos realizando un análisis de la problemática actual de TB/ VIH en el Perú, somos miembros de la UPCH y Ud ha sido identificado como un informante clave, dada su amplia experiencia en el tema y a su reconocido prestigio.
Le haremos una entrevista que consta de dos partes: la primera consta de preguntas abiertas y generales de la problemática actual de TB/VIH con la que quisiéramos conocer su opinión general y la segunda parte consta de un cuestionario específico acerca del tema.
Para su comodidad durante la entrevista no tendrá que llenar ninguna ficha, por lo que solicitamos su permiso para grabarla. Estimamos que esto nos tomará 30 minutos.
Sin más preámbulo y agradeciendo de antemano su colaboración empezaremos con la primera parte de la entrevista.

ANEXO 2:

1. En relación con TB/SIDA en el Perú:<br>¿Cuáles considera usted que son los principales problemas que existen?
2. En relación con TB/SIDA en el Perú:<br>¿Cuáles considera usted que son las soluciones a los problemas que Ud. ha planteado?
3. En relación con TB/SIDA en el Perú:<br>¿Cuáles considera usted que son las prioridades de entrenamiento o capacitación?
4. En relación con TB/SIDA en el Perú:<br>**¿Cuáles considera usted que son las principales necesidades de investigación?**<br>¿Por qué?<br>*Apuntar cada necesidad para luego preguntar porque*
5. En relación con TB/SIDA en el Perú:<br>**¿Cuáles considera usted que son las áreas prioritarias de entrenamiento relacionados a la investigación?**<br>En relación con la pregunta anterior quienes considera Ud que debería ser público objetivo del entrenamiento en investigación?<br>*Apuntar las áreas descritas*
6. ¿Qué modalidades, sistemas o formas de capacitación considera Ud que son los más adecuados para impartir entrenamiento en investigación?<br>***Ejemplos: Cursos cortos, maestrías,etc.***
7. En relación con TB/VIH en el Perú:<br>¿Cuáles considera usted que son las necesidades de entrenamiento o capacitación relacionados al manejo de los programas?
**Muchas Gracias. Hemos finalizado la primera parte de la entrevista. Estamos realizando un proyecto para que busca evaluar necesidades en capacitación en investigación clínica, operacional y de servicios de salud en TB-VIH/SIDA. Para esto estamos realizando un diagnóstico de necesidades, para luego realizar un plan estratégico de capacitación internacional que cubra dichas necesidades, nos gustaría saber:**<br>8. ¿De las preguntas realizadas, le gustaría profundizar alguna respuesta?<br><br>9. ¿Qué otra(s) pregunta(s) considera usted que debería(n) incluirse para conocer las necesidades de entrenamiento en investigación en TB/VIH en el Perú?

ANEXO 3:

INSTRUCCIONES:

Estimado doctor le vamos a plantear preguntas sobre puntos específicos de TB-SIDA, para lo cual deseamos que califique usted cada punto de acuerdo a dos parámetros:

- **la situación actual del nivel de capacitación de dicho punto en el Perú**
- **la importancia de capacitar en ese punto para solucionar la problemática de TB/ SIDA en el Perú.**

A continuación le vamos a facilitar los niveles de puntuación de cada parámetro a medir:

- VALORAR SEGÚN CRITERIO PERSONAL DEL 1 AL 6, EL **NIVEL DE CAPACITACION ACTUAL** EN EL PERÚ EN RELACIÓN A TB/SIDA.

  1 MUY POCA CAPACITACION ACTUAL
  2 POCO CAPACITACION ACTUAL
  3 MEDIANA CAPACITACION ACTUAL
  4 ALTA CAPACITACION ACTUAL
  5 MUY ALTA CAPACITACION ACTUAL
  6 NO TENGO UNA OPINIÓN DEFINIDA AL RESPECTO

- **VALORAR SEGÚN CRITERIO PERSONAL, DEL 1 AL 6 DE ACUERDO A LA IMPORTANCIA DE CAPACITACÍON.**

  1 MUY IMPORTANTE
  2 POCO IMPORTANTE
  3 MEDIANAMENTE IMPORTANTE
  4 ALTAMENTE IMPORTANTE
  5 MUY ALTAMENTE IMPORTANTE
  6 NO TENGO UNA OPINIÓN DEFINIDA AL RESPECTO

	Nivel Actual	Importancia de capacitación
Fundamentos para investigación TB/VIH		
**Principios de Epidemiología**		
**Principios de inmunología**		
**Principios de Neumología**		
**Principios de Enfermedades infecciosas**		
**Principios de Bioestadística**		
**Principios de la patogénesis bacteriológica**		
**Principios de la patogénesis viral**		
**Principios de Economía en Salud**		
**Otros**		

Desarrollo de la Investigación		
**Análisis críticos de la literatura científica**		
**Desarrollo de protocolos de investigación**		
**Sistema de Manejo de Datos**		
**Administración de recursos financieros para investigación**		
**Otros**		

Financiamiento/Publicación/Presentación		
**Estrategias para escribir, identificar y concursar para financiamiento**		
**Publicación y presentación de investigaciones**		
**Comunicación efectiva nacional entre investigadores para la toma de decisiones políticas.**		
**Otros**		

Liderazgo y trabajo en Equipo		
**Otros**		

Implementación, planificación y evaluación de Programas		
**Evaluación de Programas**		
**Planificación estratégica y desarrollo de los objetivos y actividades**		
**Técnicas rápidas de diagnóstico de necesidades**		
**Recopilación de datos y análisis para supervisores de programas**		
**Análisis situacional**		
**Manejo de recursos financieros**		
**Otros**		

ANEXOS

INSTRUMENTOS VALIDADOS

ANEXO 1: Ficha de Datos Personales

NOMBRE: ____________________<br>**Teléfono de contacto:** ____________________<br>**Dirección electrónica:** ____________________<br>____________________	EDAD:<br>____________________**años**
CENTRO DE TRABAJO:<br>____________________<br>____________________	SEXO:<br>• **Masculino** ( )<br>• **Femenino** ( )
CARGO:<br>____________________<br>____________________<br>TIEMPO EN EL CARGO:____________________	NACIONALIDAD:<br>• PERUANA ( )<br>• EXTRANJERO ( )<br>Especificar:__________
NIVEL EDUCATIVO/PROFESIÓN(especifique)<br>• **Técnico** ( )__________<br>• **Superior** ( )__________<br>• **Maestría** ( )__________<br>• **Doctorado** ( )__________<br>• **Especialidad** ( )__________	PORCENTAJE DE TIEMPO DEDICADO A (según aplique) TB SIDA ETS<br>• **1-25%** ( ) ( ) ( )<br>• **26-50%** ( ) ( ) ( )<br>• **51-75%** ( ) ( ) ( )<br>• **76-99%** ( ) ( ) ( )<br>• **100%** ( ) ( ) ( )
INSTITUCION PRINCIPAL DE TRABAJO:<br>• **MINSA** ( )<br>• **EsSalud** ( )<br>• **ONG** ( )<br>• **Universidades u otras escuelas** ( )<br>• **Privados** ( )<br>• **Otros:** ( )__________	AÑOS DE EXPERIENCIA EN TB, SIDA (según aplique):<br>TB SIDA ETS<br>• **Ninguna** ( ) ( ) ( )<br>• **Menos de 1 año** ( ) ( ) ( )<br>• **1-2 años** ( ) ( ) ( )<br>• **3-4 años** ( ) ( ) ( )<br>• **5 o más años** ( ) ( ) ( )
OCUPACION PRINCIPAL<br>• **Epidemiología** ( )<br>• **Laboratorio** ( )<br>• **Medicina Clínica** ( )<br>• **Enfermería** ( )<br>• **Administración** ( )<br>• **Obstetricia** ( )<br>• **Tecnología Médica** ( )<br>• **Biología** ( )<br>• **Psicología** ( )<br>• **Sociología** ( )<br>• **Otros:** ( )________	AREA DE INTERES PRIMARIO:<br>• TB ( )<br>• HIV/SIDA ( )<br>• ETS ( )<br>• HTLV-1 ( )<br>• Otros____________________<br>**Especificar:**<br>• **Investigación clínica.** ( )<br>• **Investigación operacional:** ( )<br>• **Investigación Servicios de Salud:** ( )<br>• **Otros:** ____________________
¿ESTARIA INTERESADO EN APLICAR A CURSOS DE ENTRENAMINETO EN TB/SIDA DURANTE EL 2004? ¿EN UPCH?<br>• SI ( ) ( )<br>• NO ( ) ( )	¿Qué TIEMPO PODRIA DEDICARLE A UN CURSO DE ENTRENAMIENTO TB/SIDA:<br>• **( ) Cursos Cortos** __________**días**<br>__________**semanas**<br>__________**meses**<br>• **( ) Diplomados** **6 meses** ( ) **1 año** ( ) **Otro** ( ) ____**meses**<br>• **( ) Maestrías** **1 año** ( ) **2 años** ( ) **Otro** ( )______**años**

De acuerdo a su experiencia, cual considera usted que son las dificultades o impedimentos para entrenarse o capacitarse:

____
____
____
____
____
____
____

De acuerdo a su experiencia, cual considera usted que son las facilidades que recibe para entrenarse o capacitarse :

____
____
____
____
____
____
____
____

Valore del 1 al 5 de acuerdo a su nivel de conocimiento, en relación a cada uno de los temas listados, donde:

1 NULO O POCO NIVEL DE CONOCIMIENTOS
2
3
4
5 EXCELENTE NIVEL DE CONOCIMIENTO

Fundamentos de ciencias para investigación en VIH/SIDA	1	2	3	4	5
Temas de investigación de VIH/SIDA	1	2	3	4	5
Tópicos de Epidemiología	1	2	3	4	5
Desarrollo y metodologías de investigación	1	2	3	4	5
Ética en investigación	1	2	3	4	5
Investigación en temas de cambio de comportamiento	1	2	3	4	5
Implementación, planificación y evaluación de programas	1	2	3	4	5
Temas de Liderazgo	1	2	3	4	5
Financiamiento/Publicación/Presentación de investigaciones	1	2	3	4	5

Podría usted señalar si ha realizado alguna investigación en VIH/SIDA durante los últimos 5 años:

SI	NO

Solo si la respuesta fue *SI*, mencionar:

TITULO	¿Ha sido Publicado?:		¿Dónde fue publicado?
	SI	NO	
	SI	NO	
	SI	NO	
	SI	NO	
	SI	NO	
	SI	NO	
	SI	NO	
	SI	NO	

ANEXO 2:

INSTRUCCIONES PARA EL DESARROLLO DEL CUESTIONARIO

Estimado colega le vamos a plantear preguntas sobre puntos específicos de VIH/SIDA. para lo cual deseamos que califique usted cada punto de acuerdo a dos parámetros:

- El actual nivel de capacitación del tópico en mención, desde su punto de vista.
- El nivel de importancia de desarrollar cada tópico en programas de capacitación, para solucionar la problemática de VIH/SIDA en el Perú.

A continuación le vamos a facilitar los niveles de puntuación de cada parámetro a medir:

- VALORAR SEGÚN CRITERIO PERSONAL DEL 1 AL 6, EL **NIVEL DE CAPACITACION** EN EL PERÚ EN RELACIÓN A VIH/SIDA.

1	NULA O ESCASA CAPACITACION
2	POCA CAPACITACION
3	MEDIANA CAPACITACION
4	ADECUADA CAPACITACION.
5	EXCESIVA CAPACITACION
NA	NINGUNA DE LAS ANTERIORES

- VALORAR SEGÚN CRITERIO PERSONAL, DEL 1 AL 6 DE ACUERDO A **LA IMPORTANCIA DE DESARROLLAR CAPACITACIÓN EN CADA TÓPICO,** EN EL PERU Y EN RELACIÓN A VIH/SIDA.

1	SIN IMPORTANCIA
2	POCO IMPORTANTE
3	REGULARMENTE IMPORTANTE
4	IMPORTANTE
5	MUY IMPORTANTE
NA	NINGUNA DE LAS ANTERIORES

CUESTIONARIO

Por favor marque con un aspa de acuerdo a su criterio

• VALORAR SEGÚN CRITERIO PERSONAL DEL 1 AL 6, EL **NIVEL DE CAPACITACION** EN EL PERÚ EN RELACIÓN A TB/SIDA.<br><br>1 NULA O ESCASA CAPACITACION<br>2 POCA CAPACITACION<br>3 MEDIANA CAPACITACION<br>4 ADECUADA CAPACITACION.<br>5 EXCESIVA CAPACITACION<br>NA NINGUNA DE LAS ANTERIORES	• VALORAR SEGÚN CRITERIO PERSONAL, DEL 1 AL 6 DE ACUERDO A LA **IMPORTANCIA DE CAPACITACÍON**.<br><br>1 SIN IMPORTANCIA<br>2 POCO IMPORTANTE<br>3 REGULARMENTE IMPORTANTE<br>4 IMPORTANTE<br>5 MUY IMPORTANTE<br>NA NINGUNA DE LAS ANTERIORES

	Nivel de Capacitación						Importancia de Capacitar						
**Fundamentos para investigación TB/VIH**	**1**	**2**	**3**	**4**	**5**	**NA**	**1**	**2**	**3**	**4**	**5**	**NA**	**Si marco N.A. explique porque:**
**Principios de Epidemiología**	☐	☐	☐	☐	☐	☐	☐	☐	☐	☐	☐	☐	________
**Principios de Inmunología**	☐	☐	☐	☐	☐	☐	☐	☐	☐	☐	☐	☐	________
**Principios de Neumología**	☐	☐	☐	☐	☐	☐	☐	☐	☐	☐	☐	☐	________
**Principios en Enfermedades Infecciosas**	☐	☐	☐	☐	☐	☐	☐	☐	☐	☐	☐	☐	________
**Principios de Bioestadística**	☐	☐	☐	☐	☐	☐	☐	☐	☐	☐	☐	☐	________
**Principios de la Patogénesis Bacteriológica**	☐	☐	☐	☐	☐	☐	☐	☐	☐	☐	☐	☐	________
**Principios de la Patogénesis Viral**	☐	☐	☐	☐	☐	☐	☐	☐	☐	☐	☐	☐	________
**Principios de Economia en Salud**	☐	☐	☐	☐	☐	☐	☐	☐	☐	☐	☐	☐	________
**Principios de Género y Sexualidad**	☐	☐	☐	☐	☐	☐	☐	☐	☐	☐	☐	☐	________
**Principios de Bioseguridad**	☐	☐	☐	☐	☐	☐	☐	☐	☐	☐	☐	☐	________
**Otros**	☐	☐	☐	☐	☐	☐	☐	☐	☐	☐	☐	☐	________
	☐	☐	☐	☐	☐	☐	☐	☐	☐	☐	☐	☐	________
	☐	☐	☐	☐	☐	☐	☐	☐	☐	☐	☐	☐	________
	☐	☐	☐	☐	☐	☐	☐	☐	☐	☐	☐	☐	________

	Nivel de Capacitación						Importancia de Capacitar						
**Tópicos en Epidemiologia y Bioestadística**	**1**	**2**	**3**	**4**	**5**	**NA**	**1**	**2**	**3**	**4**	**5**	**NA**	**Si marco N.A. explique porque:**
**Diseño de Estudios Epidemiológicos.**	☐	☐	☐	☐	☐	☐	☐	☐	☐	☐	☐	☐	________
**Análisis de Datos**	☐	☐	☐	☐	☐	☐	☐	☐	☐	☐	☐	☐	________
**Modelos matemáticos**	☐	☐	☐	☐	☐	☐	☐	☐	☐	☐	☐	☐	________
**Metodología para Vigilancia**	☐	☐	☐	☐	☐	☐	☐	☐	☐	☐	☐	☐	________
**Otros**	☐	☐	☐	☐	☐	☐	☐	☐	☐	☐	☐	☐	________
	☐	☐	☐	☐	☐	☐	☐	☐	☐	☐	☐	☐	________
	☐	☐	☐	☐	☐	☐	☐	☐	☐	☐	☐	☐	________

- VALORAR SEGÚN CRITERIO PERSONAL DEL 1 AL 6, EL **NIVEL DE CAPACITACION** EN EL PERÚ EN RELACIÓN A VIH/SIDA.

1	NULA O ESCASA CAPACITACION	4	ADECUADA CAPACITACIÓN
2	POCA CAPACITACION	5	EXCESIVA CAPACITACIÓN
3	MEDIANA CAPACITACION	NA	NINGUNA DE LASANTERIORES

- VALORAR SEGÚN CRITERIO PERSONAL, DEL 1 AL 6 DE ACUERDO **IMPORTANCIA DE CAPACITACÍON**.

1	SIN IMPORTANCIA	4	IMPORTANTE
2	POCO IMPORTANTE	5	MUY IMPORTANTE
3	REGULARMENTE IMPORTANTE	NA	NINGUNA DE LAS ANTERIORES

**Temas específicos de investigación VIH/ SIDA *(Según informante)***	**Nivel de Capacitación** 1	2	3	4	5	NA	**Importancia de Capacitar** 1	2	3	4	5	NA	**Si marco N.A. explique porque:**
Epidemiología Global de VIH/SIDA	☐	☐	☐	☐	☐	☐	☐	☐	☐	☐	☐	☐	
Epidemiología Regional de VIH/SIDA	☐	☐	☐	☐	☐	☐	☐	☐	☐	☐	☐	☐	
Inmunología del VIH	☐	☐	☐	☐	☐	☐	☐	☐	☐	☐	☐	☐	
Patología VIH	☐	☐	☐	☐	☐	☐	☐	☐	☐	☐	☐	☐	
Transmisión Perinatal del VIH	☐	☐	☐	☐	☐	☐	☐	☐	☐	☐	☐	☐	
Técnicas y Habilidades de laboratorio	☐	☐	☐	☐	☐	☐	☐	☐	☐	☐	☐	☐	
Desarrollo y prueba de Vacunas –VIH	☐	☐	☐	☐	☐	☐	☐	☐	☐	☐	☐	☐	
Profilaxis y manejo de Infecciones Oportunistas	☐	☐	☐	☐	☐	☐	☐	☐	☐	☐	☐	☐	
Terapia Antiretroviral	☐	☐	☐	☐	☐	☐	☐	☐	☐	☐	☐	☐	
Adherencia al Tratamiento	☐	☐	☐	☐	☐	☐	☐	☐	☐	☐	☐	☐	
Neurología y VIH	☐	☐	☐	☐	☐	☐	☐	☐	☐	☐	☐	☐	
Asociación con TBC	☐	☐	☐	☐	☐	☐	☐	☐	☐	☐	☐	☐	
Estigmas en VIH	☐	☐	☐	☐	☐	☐	☐	☐	☐	☐	☐	☐	
Rol de la FAMILIA/PAREJA	☐	☐	☐	☐	☐	☐	☐	☐	☐	☐	☐	☐	
Salud Mental en VIH	☐	☐	☐	☐	☐	☐	☐	☐	☐	☐	☐	☐	
Evaluación del Impacto Económico de VIH/SIDA	☐	☐	☐	☐	☐	☐	☐	☐	☐	☐	☐	☐	
Poblaciones de alto riesgo	☐	☐	☐	☐	☐	☐	☐	☐	☐	☐	☐	☐	
Otros	☐	☐	☐	☐	☐	☐	☐	☐	☐	☐	☐	☐	
	☐	☐	☐	☐	☐	☐	☐	☐	☐	☐	☐	☐	
	☐	☐	☐	☐	☐	☐	☐	☐	☐	☐	☐	☐	
	☐	☐	☐	☐	☐	☐	☐	☐	☐	☐	☐	☐	

- VALORAR SEGÚN CRITERIO PERSONAL DEL 1 AL 6, EL **NIVEL DE CAPACITACION** EN EL PERÚ EN RELACIÓN A TB/SIDA.

1 NULA O ESCASA CAPACITACION
2 POCA CAPACITACION
3 MEDIANA CAPACITACION
4 ADECUADA CAPACITACION.
5 EXCESIVA CAPACITACION
NA NINGUNA DE LAS ANTERIORES

- VALORAR SEGÚN CRITERIO PERSONAL, DEL 1 AL 6 DE ACUERDO A LA **IMPORTANCIA DE CAPACITACÍON**.

1 SIN IMPORTANCIA
2 POCO IMPORTANTE
3 REGULARMENTE IMPORTANTE
4 IMPORTANTE
5 MUY IMPORTANTE
NA NINGUNA DE LAS ANTERIORES

	Nivel de Capacitación						Importancia de Capacitar						
**Investigación en Intervención dirigida a cambios del comportamiento.**	**1**	**2**	**3**	**4**	**5**	**NA**	**1**	**2**	**3**	**4**	**5**	**NA**	**Si marco N.A. explique porque:**
**Investigación en Prevención**	☐	☐	☐	☐	☐	☐	☐	☐	☐	☐	☐	☐	
**Intervenciones para reducir factores de riesgo**	☐	☐	☐	☐	☐	☐	☐	☐	☐	☐	☐	☐	
**Teorías del comportamiento**	☐	☐	☐	☐	☐	☐	☐	☐	☐	☐	☐	☐	
**Otros**	☐	☐	☐	☐	☐	☐	☐	☐	☐	☐	☐	☐	
	☐	☐	☐	☐	☐	☐	☐	☐	☐	☐	☐	☐	
	☐	☐	☐	☐	☐	☐	☐	☐	☐	☐	☐	☐	

**Ética en investigación**	**1**	**2**	**3**	**4**	**5**	**NA**	**1**	**2**	**3**	**4**	**5**	**NA**	**Si marco N.A. explique porque:**
**Consideraciones Éticas en Investigación**	☐	☐	☐	☐	☐	☐	☐	☐	☐	☐	☐	☐	
**Investigación en poblaciones vulnerables**	☐	☐	☐	☐	☐	☐	☐	☐	☐	☐	☐	☐	
**Consentimiento Informado**	☐	☐	☐	☐	☐	☐	☐	☐	☐	☐	☐	☐	
**Otros**	☐	☐	☐	☐	☐	☐	☐	☐	☐	☐	☐	☐	
	☐	☐	☐	☐	☐	☐	☐	☐	☐	☐	☐	☐	
	☐	☐	☐	☐	☐	☐	☐	☐	☐	☐	☐	☐	

**Desarrollo de la Investigación**	**1**	**2**	**3**	**4**	**5**	**NA**	**1**	**2**	**3**	**4**	**5**	**NA**	**Si marco N.A. explique porque:**
**Análisis críticos de la literatura científica**	☐	☐	☐	☐	☐	☐	☐	☐	☐	☐	☐	☐	
**Desarrollo de protocolos de investigación**	☐	☐	☐	☐	☐	☐	☐	☐	☐	☐	☐	☐	
**Sistema de Manejo de Datos**	☐	☐	☐	☐	☐	☐	☐	☐	☐	☐	☐	☐	
**Administración de recursos financieros para investigación**	☐	☐	☐	☐	☐	☐	☐	☐	☐	☐	☐	☐	
**Otros**	☐	☐	☐	☐	☐	☐	☐	☐	☐	☐	☐	☐	
	☐	☐	☐	☐	☐	☐	☐	☐	☐	☐	☐	☐	
	☐	☐	☐	☐	☐	☐	☐	☐	☐	☐	☐	☐	

- VALORAR SEGÚN CRITERIO PERSONAL DEL 1 AL 6, EL **NIVEL DE CAPACITACION** EN EL PERÚ EN RELACIÓN A TB/SIDA.

1 NULA O ESCASA CAPACITACION
2 POCA CAPACITACION
3 MEDIANA CAPACITACION
4 ADECUADA CAPACITACION.
5 EXCESIVA CAPACITACION
NA NINGUNA DE LAS ANTERIORES

- VALORAR SEGÚN CRITERIO PERSONAL, DEL 1 AL 6 DE ACUERDO A LA **IMPORTANCIA DE CAPACITACÍON**.

1 SIN IMPORTANCIA
2 POCO IMPORTANTE
3 REGULARMENTE IMPORTANTE
4 IMPORTANTE
5 MUY IMPORTANTE
NA NINGUNA DE LAS ANTERIORES

	Nivel de Capacitación						Importancia de Capacitar						
Financiamiento/Publicación/Presentación	1	2	3	4	5	NA	1	2	3	4	5	NA	Si marco N.A. explique porque:
Estrategias para escribir, identificar y concursar por financiamiento	☐	☐	☐	☐	☐	☐	☐	☐	☐	☐	☐	☐	
Publicación y presentación de investigaciones	☐	☐	☐	☐	☐	☐	☐	☐	☐	☐	☐	☐	
Comunicación efectiva nacional entre investigadores para la toma de decisiones políticas.	☐	☐	☐	☐	☐	☐	☐	☐	☐	☐	☐	☐	
Otros	☐	☐	☐	☐	☐	☐	☐	☐	☐	☐	☐	☐	
	☐	☐	☐	☐	☐	☐	☐	☐	☐	☐	☐	☐	
	☐	☐	☐	☐	☐	☐	☐	☐	☐	☐	☐	☐	

Temas de Liderazgo	1	2	3	4	5	NA	1	2	3	4	5	NA	Si marco N.A. explique porque:
Liderazgo y trabajo en Equipo	☐	☐	☐	☐	☐	☐	☐	☐	☐	☐	☐	☐	
Otros	☐	☐	☐	☐	☐	☐	☐	☐	☐	☐	☐	☐	
	☐	☐	☐	☐	☐	☐	☐	☐	☐	☐	☐	☐	
	☐	☐	☐	☐	☐	☐	☐	☐	☐	☐	☐	☐	

Implementación, planificación y evaluación de Programas	1	2	3	4	5	NA	1	2	3	4	5	NA	Si marco N.A. explique porque:
Evaluación de Programas	☐	☐	☐	☐	☐	☐	☐	☐	☐	☐	☐	☐	
Planificación estratégica y desarrollo de los objetivos y actividades	☐	☐	☐	☐	☐	☐	☐	☐	☐	☐	☐	☐	
Diagnóstico de necesidades	☐	☐	☐	☐	☐	☐	☐	☐	☐	☐	☐	☐	
Recopilación de datos y análisis para supervisores de programas	☐	☐	☐	☐	☐	☐	☐	☐	☐	☐	☐	☐	
Análisis situacional	☐	☐	☐	☐	☐	☐	☐	☐	☐	☐	☐	☐	
Manejo de recursos financieros	☐	☐	☐	☐	☐	☐	☐	☐	☐	☐	☐	☐	
Otros	☐	☐	☐	☐	☐	☐	☐	☐	☐	☐	☐	☐	
	☐	☐	☐	☐	☐	☐	☐	☐	☐	☐	☐	☐	
	☐	☐	☐	☐	☐	☐	☐	☐	☐	☐	☐	☐	

- VALORAR SEGÚN CRITERIO PERSONAL DEL 1 AL 6, EL **NIVEL DE CAPACITACION** EN EL PERÚ EN RELACIÓN A TB/SIDA.

1 NULA O ESCASA CAPACITACION
2 POCA CAPACITACION
3 MEDIANA CAPACITACION
4 ADECUADA CAPACITACION.
5 EXCESIVA CAPACITACION
NA NINGUNA DE LAS ANTERIORES

- VALORAR SEGÚN CRITERIO PERSONAL, DEL 1 AL 6 DE ACUERDO A LA **IMPORTANCIA DE CAPACITACÍON**.

1 SIN IMPORTANCIA
2 POCO IMPORTANTE
3 REGULARMENTE IMPORTANTE
4 IMPORTANTE
5 MUY IMPORTANTE
NA NINGUNA DE LAS ANTERIORES

	Nivel de Capacitación						Importancia de Capacitar						
**Financiamiento/Publicación/Presentación**	**1**	**2**	**3**	**4**	**5**	**NA**	**1**	**2**	**3**	**4**	**5**	**NA**	**Si marco N.A. explique porque:**
**Estrategias para escribir, identificar y concursar por financiamiento**	☐	☐	☐	☐	☐	☐	☐	☐	☐	☐	☐	☐	
**Publicación y presentación de investigaciones**	☐	☐	☐	☐	☐	☐	☐	☐	☐	☐	☐	☐	
**Comunicación efectiva nacional entre investigadores para la toma de decisiones políticas.**	☐	☐	☐	☐	☐	☐	☐	☐	☐	☐	☐	☐	
**Otros**	☐	☐	☐	☐	☐	☐	☐	☐	☐	☐	☐	☐	
	☐	☐	☐	☐	☐	☐	☐	☐	☐	☐	☐	☐	
	☐	☐	☐	☐	☐	☐	☐	☐	☐	☐	☐	☐	

**Temas de Liderazgo**	**1**	**2**	**3**	**4**	**5**	**NA**	**1**	**2**	**3**	**4**	**5**	**NA**	**Si marco N.A. explique porque:**
**Liderazgo y trabajo en Equipo**	☐	☐	☐	☐	☐	☐	☐	☐	☐	☐	☐	☐	
**Otros**	☐	☐	☐	☐	☐	☐	☐	☐	☐	☐	☐	☐	
	☐	☐	☐	☐	☐	☐	☐	☐	☐	☐	☐	☐	
	☐	☐	☐	☐	☐	☐	☐	☐	☐	☐	☐	☐	

**Implementación, planificación y evaluación de Programas**	**1**	**2**	**3**	**4**	**5**	**NA**	**1**	**2**	**3**	**4**	**5**	**NA**	**Si marco N.A. explique porque:**
**Evaluación de Programas**	☐	☐	☐	☐	☐	☐	☐	☐	☐	☐	☐	☐	
**Planificación estratégica y desarrollo de los objetivos y actividades**	☐	☐	☐	☐	☐	☐	☐	☐	☐	☐	☐	☐	
**Diagnóstico de necesidades**	☐	☐	☐	☐	☐	☐	☐	☐	☐	☐	☐	☐	
**Recopilación de datos y análisis para supervisores de programas**	☐	☐	☐	☐	☐	☐	☐	☐	☐	☐	☐	☐	
**Análisis situacional**	☐	☐	☐	☐	☐	☐	☐	☐	☐	☐	☐	☐	
**Manejo de recursos financieros**	☐	☐	☐	☐	☐	☐	☐	☐	☐	☐	☐	☐	
**Otros**	☐	☐	☐	☐	☐	☐	☐	☐	☐	☐	☐	☐	
	☐	☐	☐	☐	☐	☐	☐	☐	☐	☐	☐	☐	
	☐	☐	☐	☐	☐	☐	☐	☐	☐	☐	☐	☐	
